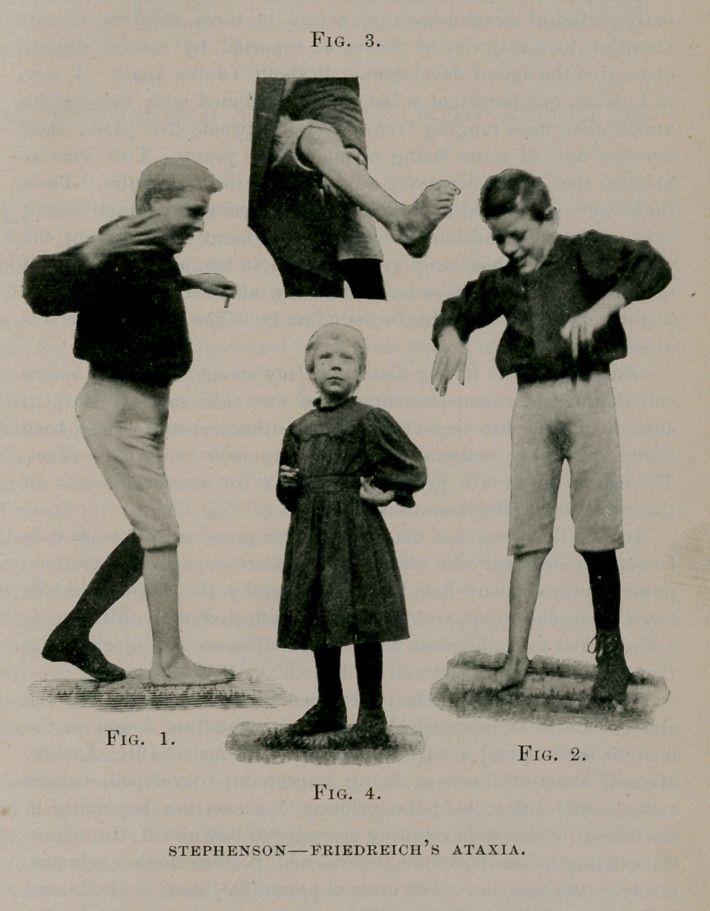# Two Cases of Friedreich’s Ataxia1Read at the twenty-ninth annual meeting of the Medical Association of Central New York, October 20, 1896.

**Published:** 1897-03

**Authors:** F. H. Stephenson

**Affiliations:** Neurologist to Woman’s and Children’s Hospital, Syracuse, N. Y.


### TWO CASES OF FRIEDREICH'S ATAXIA.[1]

By F. H. STEPHENSON, M. D.,
Neurologist to Woman's and Children's Hospital, Syracuse, N. Y.

THE following two cases of Friedreich's ataxia, whose history I will present, came under my observation through the courtesy of Dr. H. C. Merwin, of Cicero, N. Y. They are sister and brother, aged respectively six and ten years. These cases are interesting, not alone because of their rarity, only about 200 being recorded, but because of their relationship and their resemblance in certain symptoms, as well as because of their marked difference in other prominent symptoms ; yet each patient presents enough points which are typical of Friedreich's ataxia to verify the diagnosis :

CASE I.—John M., aged 10 years ; has had none of the usual diseases of infancy and childhood, except occasional attacks of indigestion and frequent colds. At birth he was apparently healthy and well developed. He was a very restless infant, but no peculiar symptoms were noticeable until he was two years of age. At that time he began to walk, which, of course, was later than the usual period. He used his arms and limbs in a very irregular and uncertain manner. He was unable to hold articles or to coördinate his arms and hands, and he constantly stumbled. Walking did not improve, as would have been the case in a child of normal development.

When he attempted to talk, which was also late, he articulated very indistinctly and with apparent difficulty. Mental development

---

1. Read at the twenty-ninth annual meeting of the Medical Association of Central New York, October 20, 1896.

was retarded, although he developed physically as rapidly as other children of his age.

*Present Condition.*—He is well nourished and looks healthy, but has a dull, heavy expression, except when attempting to do anything. On questioning him, it is plainly seen that his mentality is far below that of the average child of his age. He has never been able to attend school, and forgets quickly what he has been taught, although he appears to appreciate all that is said to him. There is a mumbling articulation and great difficulty in enunciation, which at times is explosive and blurred. Spasticity and hyperextension are well marked in the hands and arms. When walking he reminds one of a mother hen protecting her brood, as is shown in Photograph No. 1. There is also marked incoördination and over-action of the arms and fingers when using them, and it is with great difficulty that he can pick up anything from the floor, as shown in Photograph No. 2. This may be due, in part, to fear of falling, as he easily loses his balance when standing or walking. He presents a shambling and uncertain figure, slightly leaning to the left; this is probably due in part to a developing scoliosis with the convexity to the right.

On attempting to arise from a stooping to an erect position, he climbs up himself, so to speak, taking hold of his limbs in different places, reminding one of a patient affected with pseudo hypertrophic paralysis. There is marked swaying of the body during these motions.

The cranium presents several indentations; one especially is quite marked over the anterior fontanelle. There is slight but plainly marked ptosis; the pupils, though sluggish, respond to light and accommodation; the outlines are normal and there is no mystagmus. His palate is high-arched. The teeth are irregular, jagged and rapidly decaying.

He staggers and easily falls, cannot balance himself on one foot, and sways when standing with the eyes closed and arms extended. There is adduction of the feet, which he keeps well separated, and an inclination to stand on the outer side of the foot; also a partial fixation of the joints and prominence of the outer ankle bones, showing a beginning talipes, as is seen in Photograph No. 3. The plantar reflexes are present; the knee reflexes are very slight in the right limb and scarcely perceptible in the left. Hyperextension of the fingers and toes at the phalangeal articulations is frequently observed, though not constant. There is no hyperesthesia or anesthesia, but there is rectal and vesical incontinence. We have thus a defective coördination of wide extent.

Case II.—Mary, aged 6 years; apparently healthy at birth, but as she developed showed a nervous, excitable temperament. She was easily startled and exhibited a coarse tremor from head to foot. It was not constant, though increased on exertion or excitement. No marked psychical change.

She articulates quite distinctly and appreciates readily everything that is said. She did not walk or talk until over two years of age. The movements of her arms do not coördinate well.

There is a staring, frightened expression of the eyes ; no ptosis, but well-marked mystagmus ; the pupillary reflexes and outlines are normal ; the vision is good. Her teeth are irregular, jagged and decaying. Her gait is decidedly ataxic or swaying, though not so rolling and uncertain as that of her brother. The knee and wrist jerks are nearly absent ; plantar reflexes present ; no hyperextension of joints or sensory disturbances. She has both rectal and vesical incontinence, though the sphincters are better controlled than in Case I. There is no scoliosis (Photograph No. 4).

The parents are apparently sturdy people, of the agricultural class, aged 40 and 47 years. The father has a well-marked neuropathic constitution. There is no history obtainable of either syphilis or epilepsy. One brother and uncle were very immoral and erratic individuals ; one great-uncle is insane ; the grandmother is a healthy woman of advanced age. Neither she nor her daughters present any history or symptoms of nervous instability or other constitutional infirmities. On the maternal side there is a history of phthisis in the first and second generations removed. The father and grandfather were intemperate. The homes of this family and of the ancestry on the paternal side have always been in a malarial district.

Our patients have an older sister, aged twelve years, a child of average intelligence, who thus far presents none of the symptoms of Friedreich's ataxia. Her condition will be watched with great interest, as she is approaching the age when we most frequently observe the development of this disease. The fourth and last child born in this family was hydrocephalic, and died at birth.

The variation in the symptoms of the two cases presented does not seem anomalous when we regard the anatomical changes which have been found, post mortem, in Friedreich's disease, showing a different location in the points of degeneration or sclerosis. In Case I. the intellect was duller, articulation more blurred in character, ataxia of both upper and lower extremities more marked, scoliosis further advanced, knee-jerks absent or diminished, and beginning foot deformity ; while in Case II., mystagmus, tremor and ataxia were the prominent symptoms. In Case I., probably, the sclerosis in both the lateral and posterior columns is nearly equal, so there is a diminution of the knee-jerks, instead of an exaggeration or complete abolishment ; but in time, as the pos-

FIG. 3.

FIG. 1.

FIG. 4.

FIG. 2.

STEPHENSON—FRIEDREICH'S ATAXIA.

terior columns are more involved, the knee-jerks will probably disappear.

Pathological changes in the lateral columns alone probably account for the exaggerated reflexes sometimes reported in those cases whose history otherwise simulates Friedreich's ataxia. The early period of development of ataxia in these children attracts attention, as a majority of the cases reported by careful statisticians give the age of development at about twelve years. I saw, in London, one family of seven children afflicted with Friedreich's ataxia, their ages ranging from seven to twenty-five years, their average age of onset being about twelve years. Two were so helpless that it was necessary to strap them in their chairs. Their limbs were contracted and mottled in appearance; lateral curvature, mystagmus and hump-foot were prominent in all, except the youngest child, whose only symptom was an ataxic gait. None of their ancestry or relatives were similarly affected, nor were there histories of other neurotics in their family. The only factor was alcoholism.

*Etiology.*—The family history of my cases shows, as before stated, a neurotic temperament on the one side and a history of alcoholism and phthisis on the other, no other neuroses having been found. Possibly malarial poisoning may have had a toxic effect. The causes given are probably sufficient for the development of the condition of degeneration.

It is well known that the disease is progressive, although it is frequently stationary for years, and cases are reported where temporary improvement has been observed by the employment of Sayre's suspension apparatus. Patients affected with this disease are rendered more helpless after other illnesses or injuries, and they should therefore be well protected.

The larger number of cases reported are in America. Of the sixteen autopsies reported, the principal lesions are found in the medulla and spinal cord, the latter, according to Pitt, Aucher, Menzel, Dana and others, being imperfectly developed, slender and sometimes flattened; the primary degeneration beginning in the lumbar region and extending upward and downward; the sclerosis existing in the posterior, lateral and postero-median columns, rarely in the anterior. The crossed pyramidal tracts in the lateral columns, as well as the cerebellar and ascending antero-lateral tracts, are often sclerosed.

The changes in the brain tissues, outside of the medulla, do not seem of sufficient importance to occasion the symptoms observed. The anterior and posterior nerve roots are sclerosed, the peripheral nerves degenerated.

Recent observers claim the pathological change is a gliosis of the posterior columns, due to developmental errors, or, according to Déjerine, a neurogliar sclerosis, quite unlike the ordinary spinal sclerosis.

## Figures and Tables

**Fig. 1. Fig. 2. Fig. 3. Fig. 4. f1:**